# *NRAS*^*Q61R*^, *BRAF*^*V600E*^ immunohistochemistry: a concomitant tool for mutation screening in melanomas

**DOI:** 10.1186/s13000-015-0359-0

**Published:** 2015-07-25

**Authors:** Arnaud Uguen, Matthieu Talagas, Sebastian Costa, Laura Samaison, Laure Paule, Zarrin Alavi, Marc De Braekeleer, Cédric Le Marechal, Pascale Marcorelles

**Affiliations:** Inserm, U1078, Brest, F-29200 France; CHRU Brest, Service d’anatomie et cytologie pathologiques, Brest, F-29220 France; Université Européenne de Bretagne, Rennes, France; Faculté de Médecine et des Sciences de la Santé Université de Brest, EA4685, Brest, F-29200 France; CHRU Brest, Laboratoire de Génétique Moléculaire et d’Histocompatibilité, Brest, F-29220 France; Inserm, CIC1412, Brest, F-29200 France; CHRU Brest, Laboratoire de Cytogénétique et Biologie de la Reproduction, Brest, F-29220 France; Department of Pathology, University Hospital Morvan, 2, Avenue Foch 29609, Brest, France; Department of Molecular Genetics and Histocompatibily, University Hospital Morvan, 2, Avenue Foch 29609, Brest, France

**Keywords:** Melanoma, *BRAF*, *NRAS*, Molecular analysis, Immunohistochemistry

## Abstract

**Background:**

The determination of *NRAS* and *BRAF* mutation status is a major requirement in the treatment of patients with metastatic melanoma. Mutation specific antibodies against NRAS^Q61R^ and BRAF^V600E^ proteins could offer additional data on tumor heterogeneity. The specificity and sensitivity of NRAS^Q61R^ immunohistochemistry have recently been reported excellent. We aimed to determine the utility of immunohistochemistry using SP174 anti-NRAS^Q61R^ and VE1 anti-BRAF^V600E^ antibodies in the theranostic mutation screening of melanomas.

**Methods:**

142 formalin-fixed paraffin-embedded melanoma samples from 79 patients were analyzed using pyrosequencing and immunohistochemistry.

**Results:**

23 and 26 patients were concluded to have a NRAS-mutated or a BRAF-mutated melanoma respectively. The 23 *NRAS*^*Q61R*^ and 23 *BRAF*^*V600E*^-mutant samples with pyrosequencing were all positive in immunohistochemistry with SP174 antibody and VE1 antibody respectively, without any false negative. Proportions and intensities of staining were varied. Other *NRAS*^*Q61L*^*, NRAS*^*Q61K*^*, BRAF*^*V600K*^ and *BRAF*^*V600R*^ mutants were negative in immunohistochemistry. 6 single cases were immunostained but identified as wild-type using pyrosequencing (1 with SP174 and 5 with VE1). 4/38 patients with multiple samples presented molecular discordant data. Technical limitations are discussed to explain those discrepancies. Anyway we could not rule out real tumor heterogeneity.

**Conclusions:**

In our study, we showed that combining immunohistochemistry analysis targeting NRAS^Q61R^ and BRAF^V600E^ proteins with molecular analysis was a reliable theranostic tool to face challenging samples of melanoma.

**Electronic supplementary material:**

The online version of this article (doi:10.1186/s13000-015-0359-0) contains supplementary material, which is available to authorized users.

## Background

In the last decade, an improved understanding of the genetic mutations in melanoma has resulted in a better knowledge and treatment of this malignant disease. Several mutations have been identified that might affect downstream signaling to increase cell proliferation and to decrease apoptosis [1]. The mitogen-activated protein kinase (MAPK) pathway represents a major signaling cascade driving cell proliferation, differentiation and survival. This RAS-RAF-MEK-ERK pathway is constitutively activated in melanomas harboring mutations in oncogenes such as *BRAF* and *NRAS*, respectively mutated in about 50 % and 15 % of these tumors [2].

The development of targeted therapies such as selective BRAF inhibitors has improved the response rate, the progression-free survival and the overall survival of patients with metastatic *BRAF*-mutant melanomas [3–6]. The vast majority of these serine/threonine protein-kinase mutations are characterized by the substitution of valine at amino acid position 600, referred to as *BRAF*^*V600*^. This substitution leads to a conformational change resulting in constitutive kinase activity and phosphorylation of downstream targets. Concerning *BRAF*^*V600*^ mutations, about 85–90 % result in a substitution of a valine by a glutamic acid (*BRAF*^*V600E*^). Other less frequent mutations are: *BRAF*^*V600K*^ (ranging from 5 to 30 %), and *BRAF*^*V600R*^*, BRAF*^*V600D*^, *BRAF*^*V600M*^, *BRAF*^*V600E2*^, *BRAF*^*V600EK601del*^ . Other *BRAF* hot spots, such as *BRAF*^*L597P*^ and *BRAF*^*L597S*^ have incidences less than 1 %. Response to targeted-therapies concerning the most frequent and rarer mutations have been reported [7–10]. BRAF inhibitors are now the first-line treatment for patients with unresectable or metastatic melanoma which test is positive for *BRAF*^V600^, in accordance with the recommendations of the health authorities such as the National Comprehensive Cancer Network and European guidelines [11, 12].

However, targeting BRAF alone doesn’t definitively stop disease progression. Other MAPK pathway proteins such as MEK or NRAS are now the targets of new agents that are tested in a growing number of clinical trials. These new agents could increase BRAF-inhibitors’ effectiveness and hinder drug resistance [5, 13–17].

*NRAS* mutations, were classically reported to be nearly mutually exclusive to *BRAF* mutations, at least at the level of single cells, with only rare recently reported exceptions [18–22]. The main *NRAS-*mutations affect the glutamine at the amino-acid position 61, in 80–90 % of *NRAS-*mutant melanomas, with mutations encoded as *NRAS*^*Q61R*^, *NRAS*^*Q61L*^, and *NRAS*^*Q61K*^. Other less frequent mutation hot spots at amino acid 12 and 13 have been described [23]. *NRAS*^*Q61R*^ appears to be the more frequent NRAS mutation in melanoma with about 40–67 % to of *NRAS* mutations [20, 24]. *NRAS* targeting is a new field in melanoma treatment and there is no consensus on the *NRAS* inhibitors to date [25–28]. Nevertheless, the determination of *NRAS* mutational status is already of interest in melanoma treatment strategies. *NRAS* mutations are common mechanisms of resistance during treatment with BRAF inhibitors [16, 29]. More recently, therapeutic trials reported an activity of MEK1/2 inhibitors in patients with *NRAS*-mutated melanoma [30]. Recent data suggested that *NRAS* mutation in melanoma was also a predictive factor for response to high-dose interleukin 2 indicating that immunotherapy could become the first-line treatment for *NRAS*-mutated metastatic melanomas, prior to MEK inhibition [31, 32].

For these reasons, the determination of *BRAF* and *NRAS* mutation status appears to be a major criterion for treatment choices. Validated molecular methods are available to analyze this status, such as pyrosequencing technology [33–36]. However, for immunohistochemistry (IHC), mainly BRAF^V600E^ detection is yet accepted [33, 37–43]. To our knowledge, there are only two recent studies concerning anti-NRAS^Q61R^ IHC screening in the literature [19, 20]. This new antibody may provide additional information on *BRAF* and *NRAS* mutational status, especially concerning potential intratumoral genetic heterogeneity.

This context prompt us, first, to analyze, with pyrosequencing and IHC, *NRAS*^*Q61R*^, *BRAF*^*V600E*^ and other usual mutations, out of 142 primary and metastatic melanoma specimens from 79 patients, and to search for heterogeneity between primary tumors and metastases. Secondly, we attempted to evaluate the interest of this detection in the theranostic mutation screening of melanoma.

## Methods

### Case selection

We collected 142 melanoma samples from 79 patients selected from the cases analyzed at the Brest Molecular Genetic Cancer Platform (France) for theranostic purposes or archived specimens from deceased patients. In this file, some of the patients were selected because we had primary and metastatic tumoral samples and some were included because of their known *BRAF* and *NRAS* mutated status. Patients ongoing treatment with anti-BRAF target therapy were not included in our study because BRAF inhibitors can induce acquired *NRAS* mutations. So *NRAS* mutations in metastatic tumoral specimens could reflect a treatment-linked selection pressure and not true primary intra-patient tumoral heterogeneity (16;29). Cases are summarized in Table 1. The patients’ ages ranged from 17 to 90 years old (average 63.7 years old). The metastatic tumor sites were lymph nodes, skin, brain, lung, stomach, mesentery, liver and parotid gland (see Additional file 1: Table S1 for details). We analyzed both primary and metastatic formalin-fixed paraffin-embedded (FFPE) specimens for the same patient, when different samples were available. Histology slides were read to confirm the diagnosis and the presence of sufficient tumor tissue for both DNA extraction and pyrosequencing and for IHC analysis. The presence and amount of melanin-pigmentation were quantified at low magnification using a semi-quantitative scoring: 0 (absence), 1+ (less than 25 % of pigmented tumor cells), 2+ (25–49 % of pigmented tumor cells), 3+ (50–74 % of pigmented cells) or 4+ (75–100 % of pigmented tumor cells). This study was approved by CHRU Brest our institutional review board (CPP n° DC – 2008 – 214).

**Table 1:** Summary of the samples available concerning the 79 patients included in the study

Both primary tumor and metastasis	33
More than one metastasis without primary melanoma	5
Only one metastasis without primary melanoma	26
Only primary melanoma	15

### DNA extraction

Maxwell 16 CE-IVD system (Promega corporation, Fitchburg, WI, USA) combined with the Maxwell® 16 FFPE Tissue LEV DNA Purification Kit (Promega corporation, Fitchburg, WI, USA) was used to isolate DNA from 3 series of 5 μm sections of macro-dissected tissue blocks. DNA was eluted with 100 μl of water provided by the manufacturer.

### Mutation analyses

#### Pyrosequencing

The templates (173 bp of exon 15 of *BRAF* and 124 bp of exon 3 of *NRAS* genes) were amplified using the multiplex-PCR kit (Qiagen, Courtaboeuf, France) in a 20 μl final volume containing 2 μl of the tumor DNA. The genotyping of codons 600 of *BRAF* and 61 of *NRAS* was carried out on PyroMark Q24 system (Qiagen, Courtaboeuf, France) (see Table 2 for PCR primers sequences and parameters). Nucleotide numbering was done in accordance with HGVS recommendations (www.hgvs.org/mutnomen). The reference sequences NM_004333.4 for *BRAF* gene and NM_002524.4 for *NRAS* gene were used for cDNA-based numbering, i.e. the A of the ATG translational initiation codon was ascribed as +1. Analyses were considered as non conclusive (NC) when the pyrosequencing analysis process failed.

**Table 2:** Pyrosequencing primers and parameters for genotyping the codons 600-*BRAF* and 61-*NRAS*

Gene	PCR primers sequence (Forward and Reverse 5′ → 3′)	Pyrosequencing primer	Nucleotides dispensation order
*BRAF*	Biotin-GCTTGCTCTGATAGGAAAATG	GATGGGACCCACTCCATCGAGA	GTCTACTGT
	CCACAAAATGGATCCAGACA		
*NRAS*	ACACCCCCAGGATTCTTACAGA	GACATACTGGATACAGCTGGA	TCGTATCGAGAG
	Biotin-GCCTGTCCTCATGTATTGGTC		

#### Next generation sequencing

DNA libraries were produced using the Ion AmpliSeq™ Cancer Hotspot Panel v2 (Life Technologies, Saint-Aubin, France) according to the manufacturer’s instruction. Ten bar-coded (Ion Xpress Barcodes adapters kit, Life Technologies, Saint-Aubin, France) tumor DNAs libraries were sequenced simultaneously on a 316 chip. Sequences were analyzed through the Torrent suite v4.0 for alignment and SNP-InDels detection to produce BAM and VCF output files. The data were visualized with Alamut v.2.3 software (Interactive Biosoftware, Rouen, France) to review ambiguous nucleotide positions.

### Immunohistochemistry

Immunohistochemistry for NRAS^Q61R^ and BRAF^V600E^-mutant were performed using the monoclonal antibodies N-Ras (Q61R) (clone SP174, Spring Bioscience, Pleasanton, CA, USA) and BRAF V600E (clone VE1, Spring Bioscience, Pleasanton, CA, USA) at a dilution of 1:100. Immunohistochemistry was performed on Ventana Benchmark XT® automated slide preparation system (Roche Diagnostics, Meylan, France) using two different revelation kits, OptiView DAB IHC Detection Kit (Roche Diagnostics, Meylan, France) and ultraView Universal Alkaline Phosphatase Red Detection Kit (Roche Diagnostics, Meylan, France). A first line IHC was performed with ultraView® kit and a second line IHC was performed with both ultraView® and OptiView® kits for cases presenting discrepancies in IHC between samples collecting from the same patient or between results of molecular mutational status and IHC. The same protocols were applied to both NRAS^Q61R^ and BRAF^V600E^ IHC. Briefly, IHC was performed on 4 μm thick sections of the same FFPE material used for mutational testing. A positive control (*NRAS*^*Q61R*^ mutated melanoma metastasis or *BRAF*^*V600E*^ mutated melanoma metastasis) was included in each IHC round.

UltraView® Red detection kit was used through Ventana staining procedure included pretreatment with cell conditioner 1 (pH8) for 60 min, followed by incubation with diluted antibody at 37 °C for 32 min. Antibody incubation was followed by standard signal amplification with the Ventana amplifier kit, ultra-Wash, and counterstaining with one drop of hematoxylin for 12 min and one drop of bluing reagent for 4 min. Subsequently, slides were removed from the immunostainer, washed in water with dishwashing detergent, and mounted.

Optiview® DAB detection kit was used according to the manufacturer’s instructions.

Immunostaining was interpreted by a single trained pathologist (AU). As there is no recommended scoring system for the interpretation of this immunohistochemical analysis, we have scored the intensity of cytoplasmic immunolabelling as negative, “weak positive” or “strong positive”. In addition, the percentage of immunostained tumor cells was graded according to Busam et al.’s scoring system: 0 (negative), 1+ (positive in less than 25 % of tumor cells), 2+ (25–49 % of tumor cells), 3+ (50–74 % of tumor cells) or 4+ (75–100 % of tumor cells) [38].

## Results

Table 3 summarizes the tumors pyrosequencing and immunohistochemistry profile (*NRAS*^*Q61*^ or a *BRAF*^*V600*^ mutation and/or a *NRAS*^*Q61R*^ or a *BRAF*^*V600E*^ positive immunohistochemistry).

**Table 3:** Immunohistochemistry characteristics of positive samples and correlation with pyrosequencing mutational status. Strong / weak describes the intensity of staining and 1+ to 4+ referrers to the percentage of stained tumor cells. WT indicates wild-type samples according to pyrosequencing data

*NRAS* ^*Q61*^ pyrosequencing	*NRAS* ^*Q61R*^		Non *NRAS* ^*Q61R*^	
IHC NRASQ61R (SP174)	Strong	Weak	Strong	Weak
4+	10	3	0	0
3+	4	1	0	0
2+	2	1	0	1 (WT)
1+	1	1	0	0
*BRAF* ^*V600*^ pyrosequencing	*BRAF* ^*V600E*^		Non *BRAF* ^*V600E*^	
IHC BRAFV600E (VE1)	Strong	Weak	Strong	Weak
4+	13	4	1 (WT)	2 (WT)
3+	3	1	0	1 (WT)
2+	0	0	0	0
1+	0	2	0	1 (WT)

### NRAS analysis

In our study, 29.1 % of the patients (23/79) were concluded to have a *NRAS*-mutated melanoma (17.7 % (14/79) *NRAS*^*Q61R*^*, 5.1 % (4/79) NRAS*^*Q61L*^*and 6.3 % (5/79) NRAS*^*Q61K*^*). NRAS*^*Q61*^ mutations were detected in 29.5 % of the samples (38/129) including 23 *NRAS*^*Q61R*^ (c.182A>G) mutations (17.8 % of the samples, 60.1 % of *NRAS*^*Q61*^ mutations), 7 *NRAS*^*Q61L*^ (c.182A>T) mutations (5.4 % of the samples, 18.4 % of *NRAS*^*Q61*^ mutations), 8 *NRAS*^*Q61K*^ (c.181C>A) mutations (6.2 % of the samples, 21.1 % of *NRAS*^*Q61*^ mutations).

**Fig. 1:**
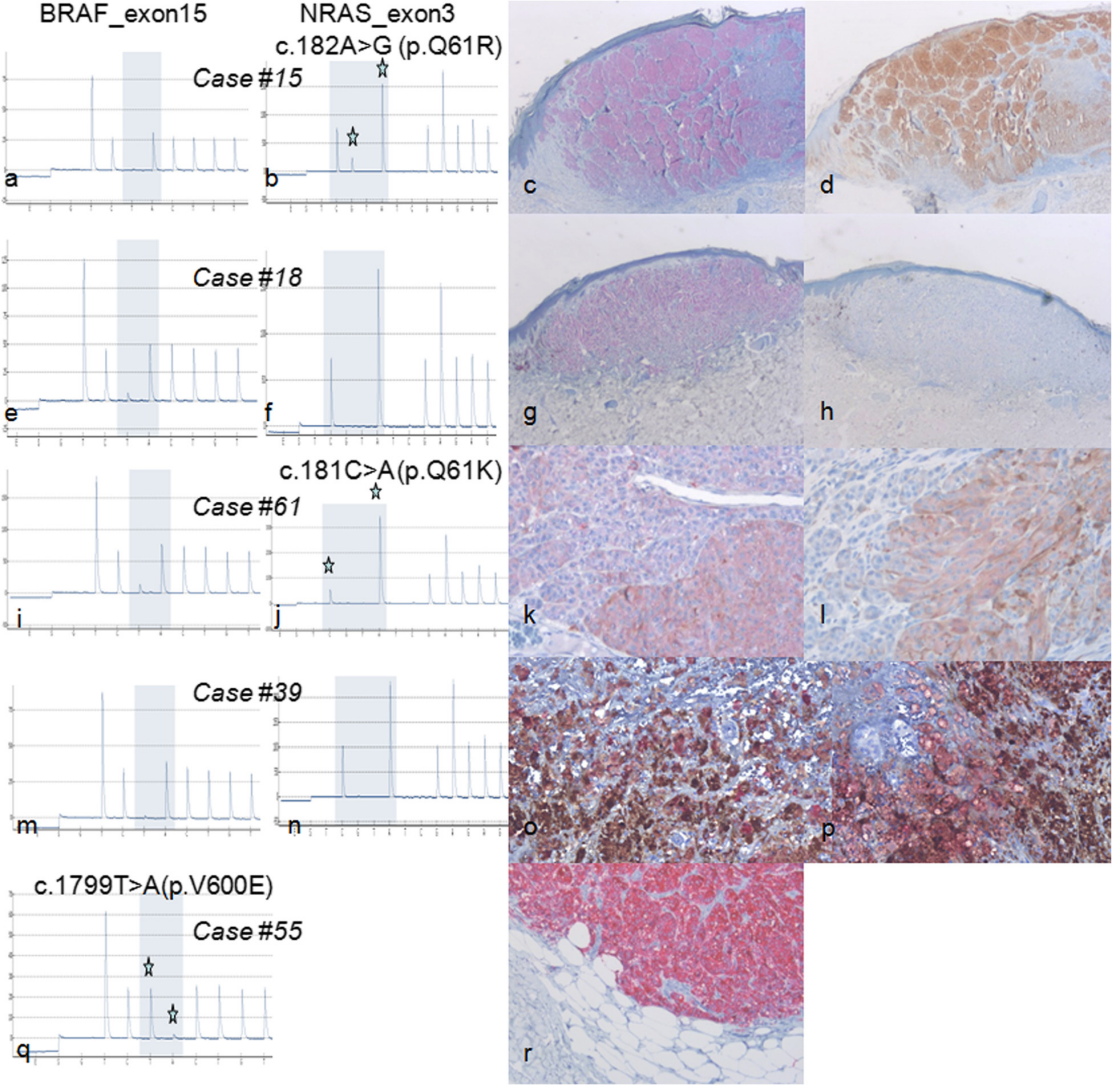
Examples of paired immunohistochemistry and pyrosequencing results. **a, b, c, d** case #15: primary *BRAF* ^*V600*^ wild-type (**a**) and *NRAS* ^*Q61R*^ mutated (**b**) melanoma with strong 4+ immunostaining with SP 174 anti-NRAS^Q61R^ *antibody* using Red (**c**) or DAB (**d**) revelation. **e, f, g, h** case #18: primary melanoma with *BRAF* ^*V600*^ (**e**) and *NRAS* ^*Q61*^ wild-type (**f**) molecular status but presenting strong 4+ staining using VE1 anti-BRAF^V600E^ antibody (**g**) and no staining with SP 174 anti-NRAS^Q61R^ antibody (**h**). **i, j, k, l** case #61: primary *BRAF* ^*V600*^ wild-type (**i**) and *NRAS* ^*Q61K*^ mutated (**j**) melanoma having moderate staining using VE1 anti-BRAF^V600E^ antibody (**k**) and fainter staining with SP 174 anti-NRAS^Q61R^ *antibody* (**l**). We concluded in a non-specific ambiguous staining in this sample with both antibodies. Note that red revelation kit as been used here as the faint melanin-pigmentation could simulate a weak DAB staining. **m, n, o, p** case #39: *BRAF* ^*V600*^ (**m**) and *NRAS* ^*Q61*^ wild-type (**n**) melanoma mesentery metastasis presenting both strong melanin pigment that could simulate a strong DAB staining and red immunostained cells with both VE1 anti-BRAF^V600E^ (**o**) and SP 174 anti-NRAS^Q61R^ (**p**) antibodies. We retrospectively concluded that all stained cells were macrophages, without any evidence of viable tumor cells in this pigmented sample. **q, r** case #55: *BRAF* ^*V600E*^ mutated (**q**) melanoma skin metastasis with strong 4+ immunostaining with VE1 anti-BRAF^V600E^ *antibody* (**r**)

All the 23 *NRAS*^*Q61R*^-mutant samples detected by pyrosequencing, had positive immunostaining, with the *NRAS*^*Q61R*^ SP174 antibody (from 1+ to 4+, and from weak to strong) (see Table 3 and Fig. 1a–d. There was one *NRAS*^*Q61R*^-wild-type sample who had positive immunostaining (2+, weak). The 7 *NRAS*^*Q61L*^-mutant and the 8 *NRAS*^*Q61K*^-mutant detected by pyrosequencing, were negative for immunostaining, with the *NRAS*^*Q61R*^ SP174 antibody.

Sensitivity of IHC with the *NRAS*^*Q61R*^ SP174 antibody was 100 % and the specificity was 99.1 %.

The immunostained areas with anti-NRAS^Q61R^ antibody were 4+ in 13/23 (56.5 %) of the samples including 10 samples with a strong staining intensity. 2/23 (8.7 %), which had a weak staining, with a 1+ or 2+ grade, were metastatic samples. In one sample (1/23, 4,3 %), about two thirds of the tumor surface/cells was weakly stained. The intensity of staining was strong but concerned less than 75 % of the tumor surface/cells (inferior to 4+) in 7/23 (30,4 %) of the samples.

A non-specific positive extra-tumoral staining was observed in 23/142 (16.2 %) samples in monocytes/macrophages cells (Fig. 1m–p). Within those 23 non specific cases, 7 samples had an adjacent tumor with *NRAS*^*Q61R*^ mutation and positive IHC.

### BRAF analysis

In our study, 32.9 % of the patients (26/79) were concluded to have a *BRAF*-mutated melanoma (22.8 % (18/79) *BRAF*^*V600E*^*, 6.3 % (5/79) BRAF*^*V600K*^*and 3.7 % (3/79) BRAF*^*V600R*^*).*

*BRAF*^*V600*^ mutations were found in 30.8 % (37/120) of the tested samples, including 23 *BRAF*^*V600E*^ (c.1799T>A) mutations (19.2 % of the samples, 62.1 % of *BRAF*^*V600*^ mutations), 8 *BRAF*^*V600K*^ (c.1798_1799GT>AA) mutations (6.7 % of the samples, 21.6 % of *BRAF*^*V600*^ mutations), 6 *BRAF*^*V600R*^ (c.1798_1799GT>AG) mutations (5 % of the samples, 16.2 % of *BRAF*^*V600*^ mutations).

Immunohistochemical analysis detected immunolabelling with the anti-BRAF^V600E^ VE1 antibody in the 23 *BRAF*^*V600E*^-mutant samples and in 5 *BRAF*^*V600E*^-wild-type samples (see Fig. 1e–h, q, r) The *BRAF*^*V600K*^ and *BRAF*^*V600R*^ samples showed no staining, as expected. Sensitivity of IHC detection with *BRAF*^*V600E*^ antibody (clone VE1) was 100 % and specificity was 95.1 %.

The proportion of stained tumor cells, within the *BRAF*^*V600E*^-mutant samples, was graded 4+ in 17/23 (73.9 %) cases. Among these cases, the staining intensity varies from strong in 13/17 (76.4 %) to weak in 4/17 (23.5 %) (see Table 3). 2/23 *BRAF*^*V600E*^-mutant samples (8.7 %) and 1 *BRAF*^*V600E*^-wild-type sample were scored 1+ (Fig. 1i–l). A non-specific positive extra-tumoral staining of the monocytes/macrophages was observed in 52/142 (36.6 %) of the samples with the anti-BRAF^V600E^ antibody including 7 samples with adjacent tumor with *BRAF*^*V600E*^ mutation and positive IHC (Fig. 1m–p).

### Molecular analysis technical issues and melanin pigmentation

Molecular analysis of both *BRAF*^*V600*^ and *NRAS*^*Q61*^ profile was possible in 119/142 samples. Molecular testing was conclusive only for either *BRAF*^*V600*^ or *NRAS*^*Q61*^ in 1/142 sample and 10/142 samples respectively. Molecular testing was not conclusive neither for *BRAF*^*V600*^ nor *NRAS*^*Q61*^ in 12/142 samples. The surface of analyzed lesions ranged from a few square millimeters to a few square centimeters and there was no correlation between the volume of tumor on the slides used for DNA extraction and the molecular test results. In the samples with partially and fully conclusive molecular results, the average proportion of tumor cells was 70.8 % (from 2 to 100 %), and melanin-pigmentation was found in 38.4 % (50/130) of the cases.

Melanin-pigmentation was reported in less than 25 % of tumor cells which were 1+ (20.7 %), 25–50 % of tumor cells 2+ (7.7 %), 50–75 % of tumor cells 3+ (6.2 %) or in more than 75 % of tumor cells 4+ (3.8 %). Within the 23 samples with non-conclusive results of at least one molecular test, 9 samples showed melanin-pigmentation. However, less than 25 % of tumor cells were pigmented among 7 of these 9 samples.

### Immunohistochemistry analysis technical issues and melanin pigmentation

IHC technique was repeated using the high-sensitive (ultraView Red) and the very high-sensitive (OptiView DAB) detection kits for discordant patients only. The results of these additional testings were identical to the initial results. There was no difference between Red and DAB detection systems. Melanin-pigmentation appeared in some cases to have a more grayish shade than the brown color of the DAB. However, in the samples with melanin-pigmentation it was not easy to distinguish between DAB focal cytoplasmic strong or weak staining and real melanin-pigmentation of tumor cells. Unlike DAB, phosphatase alkaline Red detection allowed easier distinction between a positive staining and melanin-pigmentation. The melanin-pigmentation was present in 7/23 (30.4 %) of *NRAS*^*Q61R*^-mutant samples (i.e. mutated with pyrosequencing and SP174 antibody immunostaining) and in 12/23 (52.2 %) of the *BRAF*^*V600E*^ mutant lesions (i.e. mutated with pyrosequencing and VE1 antibody immunostaining). In contrast, 36/96 (37.5 %) of the other samples showed at least focal melanin-pigmentation (1 + and higher) with melanin-pigmentation that could simulate a DAB staining.

### Discrepancies between techniques and samples

For the samples showing non-conclusive molecular analysis, IHC was interpreted in all these cases. They were scored as positive with anti-NRAS^Q61R^ antibody in 1/13 (7.7 %) and with anti-BRAF^V600E^ antibody in 3/22 (13.6 %).

Among the 38 patients with paired (primary and metastatic tumors) multiple samples, the genotype determined by pyrosequencing was discordant in 4 patients (i.e. inter-lesions): 3 for *NRAS*^*Q61*^ (cases #39, #61, #66) and 1 for *BRAF*^*V600*^ (case #4). Furthermore, cases #39 and #66 had also discordant IHC using anti-NRAS^Q61R^ antibody. 2 other patients presented with a discordant result using anti-BRAF^V600E^ IHC (cases #48, #61). For the 4 patients having discordant genotype results (cases #4, #39, #61 and #66), microsatellite markers confirmed that the samples originated from the same individual (data not shown). See Additional file 1: Table S1 for details.

## Discussion

As BRAF inhibition became the reference treatment of *BRAF*^*V600*^-mutant metastatic melanomas, screening for *BRAF*^*V600*^ mutations was a major requirement for an optimal treatment with targeted therapies [44]. *NRAS* and *BRAF* mutations have been reported traditionally to be nearly mutually exclusive in a single tumor with nevertheless rare exceptions of double mutants [19–21]. As recent data suggested, new more efficient therapeutic options are becoming available in *NRAS*-mutated melanomas such as MEK-inhibitors and immunotherapy [26, 32]. Beside DNA based techniques, IHC with mutation-specific antibodies are emerging as a complementary theranostic tool. We evaluated the combination of novel NRAS^Q61R^ and BRAF^V600E^ immunohistochemistry as well as pyrosequencing for mutation status profiling. In our study, mutations frequencies do not reflect frequencies encountered in the general melanoma population. Nevertheless, the numerous *NRAS* mutated samples included in our study offers the opportunity to focus on performances of the new SP174 anti- NRAS^Q61R^ antibody in the melanoma mutational screening.

### Molecular analysis

The study by Colomba et al. only reported 1.8 % pyrosequencing analysis failure [33]. The relatively high DNA analysis failure rate (16 %; 23/142) reported in our study may be explained by the fact that many samples were archived samples, i.e. did not undergo standardized pre-analytical steps used for current samples. In our daily practice, the rate of DNA analysis failure in melanoma samples is about 3 % and about 1.5 % concerning non-melanoma samples (unpublished data). The reasons for DNA analysis failures are not always well-known. Here, we hypothesized that non-formalin fixatives, late or over-fixation may explain such failure. A high amount of melanin-pigmentation, which can play a polymerase chain reaction-inhibitor role, can explain several amplification failures but it is not a single sufficient factor as the molecular analysis of most pigmented lesions were conclusive [45].

### Immunohistochemistry

IHC detection of NRAS^Q61R^ protein with SP174 antibody detected all (100 %) the *NRAS*^*Q61R*^-mutant samples (Fig. 1a–d). Our results are consistent with those obtained by Massi et al. and Ilie et al. who have recently reported a sensitivity of 100 % and a specificity of 100 % with this novel antibody [19, 20]. This antibody showed performances similar to those published on BRAF^V600E^ antibody [33, 37–41]. As expected, for other mutations spots/points, NRAS^Q61R^ and BRAF^V600E^ antibodies were ineffective. These mutations represented 29/142 (20.4 %) of the samples (17/79 patients, 21.5 %) in this study.

To valid IHC screening, the staining intensity must be strong enough to be distinguished from an artifact background or melanin-pigmentation. Even if melanin-pigmentation was sometimes identified as a grayish pigmentation, the red detection was, to our experience more suitable. Chen et al. have proposed the use of mild hydrogen peroxide and heating to remove endogenous melanin in high pigmented samples to improve the reliability of using anti-BRAF^V600E^ immunohistochemistry with DAB staining [46]. Furthermore, melanin-pigmentation was also present in monocytes/macrophages called melanophages. Distinction between a tumor cell and a melanophage was difficult, e.g. in case #39 where the pigmented macrophages could not be distinguished from DAB-detected immunostained tumor cells (Fig. 1m–p).

In our study, some lesions were regarded as difficult to analyze, along with difficulties reported in previous studies on anti- BRAF^V600E^ IHC whatever chromogens was used [35, 37, 38, 40–43, 46, 47]. Such ambiguous staining images may explain the positive IHC scoring of *BRAF*^*V600E*^-wild-type samples (cases #48 and #61). The positivity of these two cases was finally considered to be an artifact background, even more in case #61 where there was also a very weak staining with anti-NRAS^Q61R^ antibody (Fig. 1i–l). Interpretation issues of NRAS^Q61R^ IHC have also recently been reported by Ilie et al. who have finally considered a faint staining as non specific and pointed out the need of a moderate to strong cytoplasmic staining of at least 60 % of tumor cells to consider this IHC as positive [19]. Massi et al. have reported a double mutant *BRAF*^*V600E*^ and *NRAS*^*Q61R*^ strongly stained with the two anti- BRAF^V600E^ and anti-NRAS^Q61R^ antibodies [20]. Another limitation of IHC was the interpretation of non-specific staining of monocytes/macrophages that can be interspersed or clustered, i.e. representing a focal false positive IHC scoring (Fig. 1m–o). These limitations were similar for both NRAS^Q61R^ and BRAF^V600E^ IHC analysis. In contrast with the literature, none of our tested samples with necrosis (some metastatic samples of this study) were misidentified nor showed altered IHC results. One of the two published studies to date concerning anti-NRAS^Q61R^ antibody have also reported those issues [19].

### Tumor heterogeneity or technical artifacts?

IHC of the specimens gave various intensities and percentages of tumor cells in paired primary and metastatic samples, even though they were positive with pyrosequencing. There was no difference in the staining distribution between primitive or secondary lesions. Only two cases (cases #66 and #70) seemed to have discordant primitive and secondary data. In case #66, the primary sample contained only about 2 % tumor cells and IHC using antibody targeting NRAS^Q61R^ allowed the identification of the mutated protein whereas pyrosequencing failed to identify the mutation. In the same case, an in-transit metastasis was not stained with NRAS^Q61R^ antibody and was non-conclusive for both NRAS^Q61^ and BRAF^V600^ while both lymph node and skin metastasis were strongly NRAS^Q61R^ stained and mutated. In case #70, the primitive nodular melanoma was stained with BRAF^V600E^ antibody but non-conclusive using molecular analysis and the brain metastasis were *BRAF*^*V600E*^ stained and mutated. In this same case #70, a third lesion (a lymph node metastasis) was not stained and was non-conclusive for molecular analysis. Although we cannot rule out samples inappropriate pre-analytical features which may explain both IHC false negatives and the failure of pyrosequencing, these findings are consistent with real tumoral heterogeneity. Tumoral heterogeneity was also found in previous studies [19, 20, 38, 48–50].

Nevertheless such heterogeneity did not seem to provide a major explanation for the discrepancies among a patient’s samples. Allelic detection limits should also be taken into account for pyrosequencing false negatives.

The small proportion of tumor cells compared to non-tumor cells in some samples may be an explanation to this discrepancy as illustrated above by case #66. Moreover in case #4, in regard to *BRAF*^*V600*^ mutational status, the lymph node sample contained only about 10 % of tumor cells on its histopathological section and was identified as *BRAF*^*V600E*^*-*wild-type whereas other samples containing 40 and 70 % of tumor cells showed a clear identification of *BRAF*^*V600E*^ mutation. An additional ultra-deep sequencing indeed identified 8 of 337 allelic copies (2.3 %) presenting a c.1799T>A mutation (p.V600E) in the same DNA sample used for pyrosequencing. This 2.3 % rate of mutated alleles is undoubtedly bellow pyrosequecing detection threshold. Consequently, ultra-deep sequencing can be used as an ancillary identification tool to clarify discrepancies in samples with a low density of tumor cells. A good correlation has already been described between anti-BRAF^V600E^ IHC and ultra-deep sequencing of *BRAF* in colorectal carcinomas and in melanomas. Ihle et al. also reported a 3 % rate of mutated alleles in a pyrosequencing-negative but IHC BRAF^V600E^-positive sample [51, 52]. Mutated tumor cell sample (intended for DNA extraction) enrichment by macro- or micro-dissection guided by IHC, may improve the identification process.

Both tumor and technical features must probably be considered to explain apparent intratumoral heterogeneity in our and previous studies in contrast with others reporting strong intratumoral homogeneity [37, 53].

### How to manage samples with unclear staining?

Analysis of the available literature showed that most studies report conclusive data on *BRAF*^*V600*^ mutation status, with only a few that reported non conclusive cases for molecular and IHC analysis [33, 37]. In the study by Boursault et al., 3 cases remained unclear in regard to VE1 anti-BRAF^V600E^ immunostaining because of a faint equivocal brown staining in a BRAF^V600E^-wild-type primary melanoma, a BRAF^V600E^-mutant primary melanoma and a lymph node BRAF^V600K^-mutant metastasis [37]. Busam et al. also reported cases of 1+ weak stained BRAF^V600E^-mutant lesions with this antibody but regarded these cases as interpretable positive IHC cases [38]. On the contrary, Heinzerling et al. and Ihle et al. regarded a weak staining as negative [9, 51]. On 111 cases, Colomba et al. regarded 8 (7.2 %) cases as equivocal for BRAF^V600E^ IHC because of stained macrophages, i.e. consistent with our study, and because of a nuclear staining instead of a cytoplasmic one [33]. In our study, we did not observe any isolated nuclear staining. Interpretation criteria for unclear IHC results are not known [9, 33, 37, 38, 51]. In our study, 6 *NRAS*^*Q61R*^*-*mutant and 1 *NRAS*^*Q61*^-wild-type samples were weakly stained whereas 7 *BRAF*^*V600E*^ -mutant and 5 *BRAF*^*V600*^-wild-type samples also presented a weak positivity (Table 3). According to our data, caution must be exercised in case of unclear weak staining. We note that in our experience there are less numerous unclear cases with anti-NRAS^Q61R^ antibody than with anti-BRAF^V600E^ one. Massi et al. also have reported moderate to strong cytoplasmic staining with anti-NRAS^Q61R^ antibody in all their 14 *NRAS*^*Q61R*^*-*mutated samples [20]. Ilie et al. have required a moderate to strong staining in more than 60 % of tumor cells to consider the anti-NRASQ61R IHC as positive [19]. In this manner, our faintly stained- but nevertheless NRAS^Q61R^-mutated samples above mentioned would have been scored negative in their study. This difference points out the need of homogeneous technical interpretative criteria in this field of mutation-specific IHC. To our opinion, both molecular and IHC analysis have to be taken into account to conclude in a mutated of not mutated *NRAS* and *BRAF* status in these samples.

### Discrepancies between molecular and IHC analysis

Discrepancies between IHC and molecular data were also reported within a same sample. As described in our study, Massi et al. have encountered 3 discordant cases concerning 2 samples initially considered as *NRAS*^*Q61R*^ mutated but non-stained with SP-174 antibody and, at the opposite, 1 sample positive with this antibody but wild-type concerning molecular analysis. Additional molecular genetic analysis have permitted to correct this discrepancies with the final identification of two *NRAS*^*Q61K*^ mutations in the 2 negative samples with IHC, and with the identification of a *NRAS*^*Q61R*^ mutation in the IHC positive sample [20]. Ilie et al. finally have not encountered discrepant cases by considering high stringent interpretative cut off (i.e. moderate to strong staining of at least 60 % of tumor cells) and have reported a specificity of 100 % of anti-NRAS^Q61R^ IHC on the basis of pyrosequencing NRAS mutational status. The specificity of their assay was diminished to 92 % taking into account less stringent criteria (i.e. weakly stained samples corresponding to false positive as *NRAS* wild-type or other than *NRAS*^*Q61R*^-mutated samples) [19].

Concerning BRAF^V600E^ mutation, Feller et al. reported a case of a lesion being positive with IHC and negative with pyrosequencing analysis of BRAF^V600E^ [40]. Chen et al. reported a similar case in an esophageal tumor [46]. These results concurred with those of Marin et al. for faint to moderate stained lesion [42]. Analyzing circulating melanoma cells, Hofman et al. reported 15 % of samples to be IHC positive and pyrosequencing negative [47]. Lade-Keller et al. reported on the one hand, 4/13 cases IHC positive and COBAS® system negative for BRAF mutation. These authors, on the other hand, reported a case of *BRAF*^*V600E*^ mutated melanoma positive on COBAS® but negative on IHC [35]. Similar IHC negative and *BRAF*^*V600E*^ mutated samples were also reported by Skorokhod et al. in 2/14 cases and by Ihle et al. who reported a double mutated codons 600 and 601 tumor, negative using IHC [43, 51]. Taking into account the molecular data as a reference, Long et al. found 35 true positives, 2 false negatives, 57 true negatives and 3 false positives using anti-BRAF^V600E^ antibody. In Long’s study, repeated molecular analysis gave the following explanations for the false positive and negative results. One false negative was reported to be due to an initial genetic testing: false positive revealed a *BRAF*^*K601Q*^ mutation instead of a BRAF^V600E^ one. Three false positives were also regarded as related to genetic analysis failure. Finally 2 more BRAF^V600E^ mutated lesions and a third lesion did not allow molecular detection of the mutation due to their lacking of enough tumor cells. Nevertheless, one BRAF^V600E^ mutated case remained IHC negative without clear explanation [41]. Futhermore, Ihle et al. reported a case of cross reactivity with non *BRAF*^*V600E*^ mutations with an IHC positive sample using VE1 antibody whereas molecular analysis showed a *BRAF*^*V600R*^ mutation [51]. Heinzerling reported the same cross reactivity with a *BRAF*^*V600K*^ mutated sample [9]. Finally, in our study, only one sample (case #18) was strongly and homogeneously (4+) stained with VE1 anti-BRAF^V600E^ antibody but wild-type using pyrosequencing without obvious explanation to this discrepancy to date.

### Which place for mutation-specific IHC?

To our opinion, the main advantage of IHC testing is the accessibility of the test that can be realized in the same time as histopathological examination. A strong positive IHC staining quick and sure for NRAS^Q61R^ or BRAF^V600E^ antibodies can accelerate the patient’s therapeutic management. Nevertheless, conventional, longer but wider analysis using molecular techniques must be realized in case of negative or unclear IHC results. Indeed, given the performances of these IHC screenings and the need to identify all the variants of NRAS^Q61^ and BRAF^V600^ to guide treatment decision, IHC cannot replace molecular analysis, an essential step in melanoma diagnosis. But IHC can be used as a first-line or a concomitant screening tool in the management of every-day sample, especially the more challenging ones, as proposed in some studies [19, 54].

## Conclusions

The determination of the molecular mutated status of metastatic melanoma becomes mandatory on the best way to treat the patient. Intra- and inter-tumor molecular heterogeneity is a challenge on the avenue to a molecular diagnostic determination of melanoma. Technical artifacts must be the first explanation for such an apparent heterogeneity. As multiple primary melanomas can exist in a same patient, and as those primary malignant lesions can sometimes be totally regressive and unnoticed, precaution is required when selecting the sample to test for molecular status [53]. This is why we recommend a more exhaustive and systematic analysis of multiple-tumor specimens for patient’s care and management rather than a single molecular status [55]. As showed in our study, technical limitations concern both molecular and IHC analysis.

SP174 anti-NRAS^Q61R^ antibody seemed overall to have similar performances as VE1 anti-BRAF^V600E^, with only some samples remaining ambiguous. To face these challenges, we suggest a systematic combined IHC analysis of every sample, using anti-NRAS^Q61R^ and anti-BRAF^V600E^ antibodies and molecular analysis with pyrosequencing to complete IHC. We believe that the combination of these techniques and the comparison of their results may improve the theranostic strategy of melanoma by reducing each technique’s drawbacks.

## Additional file

Additional file 1: Table S1: Detailled features of cases and samples analyzed.
