# Supplementary material for: NRASQ61R, BRAFV600E immunohistochemistry: a concomitant tool for mutation screening in melanomas
Source: Diagn Pathol. 2015 Jul 25;10:121. doi: 10.1186/s13000-015-0359-0 (PMC4513673; doi:10.1186/s13000-015-0359-0)
Supplement: Additional file 1: Table S1: — Detailled features of cases and samples analyzed. [file 13000_2015_359_MOESM1_ESM.pdf]

Supplementary data: Detailled features of cases and samples analyzed.

**Case**	**Age at time of primary tumor diagnosis /Gender**	**Primitive lesion type, site and thickness (mm)/ metastatic site**	**Percentage of tumor cells in samples**	**Melanin pigmentation**	**BRAFV600 genotype (pyrosequencing)**	**IHC BRAFV600E (VE1) proportion of cells/intensity**	**NRASQ61 genotype (pyrosequencing)**	**IHC NRASQ61R (SP174) proportion of cells/intensity**
**1**	59/male	NM, face, 3,25	80	3+	NC	0	WT	0
		Lymph node	60	3+	WT	0	WT	0
**2**	41/male	Lymph node	80	0	V600E	4+, strong	WT	0
**3**	51/male	SSM, back, 2,2	80	1+	NC	0	NC	0
		Lymph node	95	0	WT	0	WT	0
		Skin	90	1+	WT	0	WT	0
**4**	75/male	SSM, back,2,9	70	1+	V600E	4+, strong	WT	0
		Lymph node	20	0	NC	4+, strong	WT	0
		Lymph node	40	0	V600E	4+, strong	WT	0
		Lymph node	10	0	WT	3+, weak	WT	0
**5**	90/female	SSM, heel, 9	95	1+	NC	0	NC	0

		Skin	90	0	NC	0	NC	0
**6**	72/male	Lymph node	90	0	WT	0	Q61R	1+, strong
**7**	68/male	SSM, ear, 1,95<br>Lung	70<br>90	0<br>0	WT<br>NC	0<br>0	WT<br>NC	0<br>0
**8**	88/female	SSM, calf, 13<br>Lymph node	90<br>80	1+<br>2+	V600E<br>V600E	4+, strong<br>4+, strong	WT<br>WT	0<br>0
**9**	75/female	NM, calf, 2,5<br>In-transit<br>In-transit	80<br>5<br>90	0<br>0<br>0	NC<br>NC<br>WT	0<br>0<br>0	NC<br>NC<br>Q61L	0<br>0<br>0
**10**	81/female	MLM, nasal<br>In transit	70<br>90	0<br>0	WT<br>WT	0<br>0	Q61K<br>Q61K	0<br>0
**11**	72/female	MLM, nasal<br>Stomach	90<br>90	0<br>0	NC<br>NC	0<br>0	NC<br>WT	0<br>0
**12**	77/male	SSM, back, 1,2<br>Lymph node<br>Mesentery	20<br>80<br>70	3+<br>4+<br>1+	WT<br>WT<br>WT	0<br>0<br>0	WT<br>WT<br>WT	0<br>0<br>0
**13**	59/male	Parotid gland	50	1+	WT	0	WT	0
**14**	54/male	Liver	25	2+	WT	0	WT	0
**15**	43/male	NM, knee, 1,8	90	0	NC	0	Q61R	3+, strong

		Local recurrency<br>Lymph node<br>Lymph node	70<br>95<br>90	0<br>0<br>0	WT<br>NC<br>WT	0<br>0<br>0	Q61R<br>NC<br>Q61R	4+, strong<br>4+, strong<br>4+, strong
**16**	86/male	Lymph node	90	0	V600E	4+, strong	WT	0
**17**	67/female	Lymph node	90	0	WT	0	Q61R	4+, strong
**18**	70/male	SSM, arm,1,2	50	0	WT	4+, strong	WT	0
**19**	79/male	SSM, flank, 5,6<br>Lymph node	80<br>90	1+<br>0	WT<br>WT	0<br>0	Q61R<br>Q61R	4+, strong<br>4+, weak
**20**	71/female	Skin	60	0	WT	0	WT	0
**21**	42/male	SSM, leg, 10	90	0	WT	0	Q61R	2+, weak
**22**	87/male	NM, face, 10,5	80	2+	WT	0	WT	0
**23**	74/male	Lymph node	80	0	WT	0	WT	0
**24**	65/male	SSM, ear,7<br>In-transit	95<br>80	0<br>0	WT<br>NC	0<br>0	WT<br>WT	0<br>0
**25**	75/female	SSM, trunk, 4<br>Lymph node<br>Lymph node<br>Lymph node	30<br>90<br>90<br>80	0<br>1+<br>0<br>0	V600R<br>V600R<br>V600R<br>V600R	0<br>0<br>0<br>0	WT<br>WT<br>WT<br>WT	0<br>0<br>0<br>0
**26**	52/male	NM, trunk, 7,1	70	0	WT	0	WT	0

**27**	55/female	Brain<br>Brain	95<br>70	0<br>0	WT<br>WT	0<br>0	WT<br>WT	0<br>0
**28**	37/male	Brain	50	4+	V600E	4+, strong	WT	0
**29**	59/male	Lymph node	50	0	WT	0	Q61R	4+, weak
**30**	50/female	Brain<br>Lung	90<br>90	0<br>0	WT<br>WT	0<br>0	Q61K<br>Q61K	0<br>0
**31**	68/female	NM, face, 2,4	80	1+	WT	0	WT	0
**32**	17/female	SSM, scalp, 2,5<br>Lymph node	30<br>80	0<br>1+	WT<br>WT	0<br>0	WT<br>WT	0<br>0
**33**	61/male	SSM,foot, 6<br>Lymph node	70<br>95	1+<br>2+	WT<br>WT	0<br>0	WT<br>WT	0<br>0
**34**	39/female	NM, back, 3,9	50	0	V600E	4+, weak	WT	0
**35**	40/female	Liver	100	1+	WT	0	WT	0
**36**	52/male	Lymph node	90	0	WT	0	Q61K	0
**37**	67/male	NM, back,12<br>Local recurrence<br>Lymph node	75<br>80<br>80	1+<br>0<br>2+	NC<br>WT<br>WT	0<br>0<br>0	WT<br>WT<br>WT	0<br>0<br>0
**38**	44/female	Skin	90	0	WT	0	WT	0
**39**	73/female	SSM, leg, 2,5	20	1+	NC	0	Q61R	3+, strong

		Mesentery	50*	4+	WT	0	WT	0
**40**	64/male	SSM, heel, 2,5	15	3+	WT	0	WT	0
		Lymph node	20	4+	WT	0	WT	0
**41**	82/male	Lymph node	95	2+	V600E	4+, weak	WT	0
		Lymph node	35	0	V600E	4+, strong	WT	0
**42**	61/male	Lung	70	1+	WT	0	Q61R	3+, strong
		Brain	40	0	WT	0	Q61R	2+, strong
**43**	49/female	NM, face, 5,5	90	0	V600E	3+, strong	WT	0
**44**	68/female	NM, face, 2,5	80	1+	V600E	4+, weak	WT	0
**45**	63/male	NM, face, 11	90	0	WT	0	WT	0
**46**	71/male	Skin	90	0	WT	0	Q61R	4+, weak
**47**	61/male	NM, back, 11	90	1+	WT	0	WT	0
		Skin	30	1+	WT	0	WT	0
		Brain	90	4+	WT	0	WT	0
**48**	56/male	DM,temple, 3,5	10	0	WT	0	WT	0
		Lymph node	95	0	WT	1+, weak	WT	0
		Lymph node	40	0	WT	0	WT	0
		Skin	80	0	NC	0	WT	0
**49**	64/male	Liver	60	3+	WT	0	WT	0

**50**	51/female	Skin	60	0	WT	0	WT	0
**51**	63/female	NM, face, 3<br>Parotid gland	60<br>20	0<br>1+	V600E<br>V600E	4+, strong<br>1+, weak	WT<br>WT	0<br>0
**52**	86/female	Local recurrence	60	0	WT	0	Q61L	0
**53**	75/male	SSM, back, 2,7<br>Lymph node	40<br>70	0<br>0	V600K<br>V600K	0<br>0	WT<br>WT	0<br>0
**54**	55/male	Primitive biopsy<br>Lymph node	50<br>80	0<br>1+	V600K<br>V600K	0<br>0	WT<br>WT	0<br>0
**55**	55/male	Skin	80	0	V600E	4+, strong	WT	0
**56**	89/female	Primitive biopsy	80	0	V600R	0	WT	0
**57**	80/male	Primitive biopsy	80	0	WT	0	Q61L	0
**58**	53/male	Primitive biopsy	90	0	V600R	0	WT	0
**59**	49/male	NM, back, 9	90	2+	WT	0	Q61R	4+, strong
**60**	68/female	NM, leg, 27<br>In-transit<br>Skin<br>Skin	90<br>90<br>80<br>70	0<br>1+<br>0<br>0	WT<br>WT<br>WT<br>WT	0<br>0<br>0<br>0	Q61L<br>Q61L<br>Q61L<br>Q61L	0<br>0<br>0<br>0
**61**	31/male	SSM, arm, 5<br>Local recurrence	90<br>10	2+<br>3+	WT<br>WT	4+, weak**<br>4+, weak**	Q61K<br>WT	0**<br>0**

		Lymph node	90	3+	WT	0	Q61K	0
**62**	81/female	Lymph node	60	0	WT	0	Q61R	4+, strong
**63**	40/male	Lymph node	90	0	V600E	4+, weak	WT	0
**64**	24/male	SSM, leg, 1,3<br>Lymph node	20<br>70	0<br>1+	NC<br>V600E	3+, weak<br>3+, strong	NC<br>WT	0<br>0
**65**	64/male	Lymph node<br>Lymph node	70<br>90	0<br>0	V600K<br>V600K	0<br>0	WT<br>WT	0<br>0
**66**	81/female	SSM, foot, 2<br>In-transit<br>Lymph node<br>Skin	2<br>70<br>90<br>80	0<br>1+<br>2+<br>0	NC<br>NC<br>WT<br>WT	0<br>0<br>0<br>0	WT<br>NC<br>Q61R<br>Q61R	2+, weak<br>0<br>3+, strong<br>4+, strong
**67**	70/female<br>74/female<br>75/female	SSM arm 2,2<br>In-transit<br>Skin<br>Skin<br>Skin	50<br>70<br>80<br>20<br>80	1+<br>0<br>0<br>0<br>0	WT<br>WT<br>WT<br>WT<br>WT	0<br>0<br>0<br>0<br>0	Q61R<br>Q61R<br>Q61R<br>Q61R<br>Q61R	2+, strong<br>3+, weak<br>4+, strong<br>4+, strong<br>4+, strong
**68**	60/female	Lymph node	60	0	V600E	4+, strong	WT	0
**69**	50/female	NM, nail, 10<br>Skin	90<br>95	1+<br>0	NC<br>WT	0<br>0	NC<br>NC	0<br>0

		In-transit<br>Lymph node	90<br>90	0<br>0	WT<br>WT	0<br>0	WT<br>WT	0<br>0
**70**	56/male	NM, neck, 2,6<br>Lymph node<br>Brain	70<br>90<br>90	1+<br>3+<br>2+	NC<br>NC<br>V600E	2+, strong<br>0<br>1+, weak	WT<br>NC<br>WT	0<br>0<br>0
**71**	35/female	Lymph node	100	0	WT	0	Q61K	0
**72**	61/male	SSM, back, 12,4<br>Lymph node	80<br>80	1+<br>3+	V600E<br>V600E	4+, strong<br>3+, weak	WT<br>WT	0<br>0
**73**	88/female	Lymph node	90	0	WT	0	WT	0
**74**	76:male	MLM, anus<br>Lymph node<br>Lymph node	60<br>80<br>95	1+<br>0<br>0	WT<br>WT<br>WT	0<br>0<br>0	WT<br>WT<br>WT	0<br>0<br>0
**75**	77/male	Skin	70	0	V600K	0	WT	0
**76**	63/male	Lymph node	90	0	V600E	4+, strong	WT	0
**77**	83/male	Skin	80	1+	WT	0	Q61R	1+, weak
**78**	64/male	Lung	70	1+	V600E	3+, strong	WT	0
**79**	60/female	NM, trunk,3	80	0	V600K	0	WT	0

* This metastatic sample has been read back because of discrepancies between the two samples of a single patient. In fact, this metastatic sample only consisted in strong pigmented macrophages without obvious viable tumor cells.

** These samples have been read back because of discrepancies between weak positve IHC with $BRAF^{V600E}$ antibody and *NRAS*^{Q61K} mutation status. Finally, a very weak staining with $NRAS^{Q61R}$ antibody was also noted, considered initially as non significative. The weak to very weak intensity of both of $BRAF^{V600E}$ and $NRAS^{Q61R}$ IHC as finally been considered as non specific.

DM: Desmoplastic Melanoma; MLM: Mucosal Lentiginous Melanoma; NM: Nodular Melanoma; SSM: Superficial Spreading Melanoma; WT : wild-type; NC: Non Conclusive.

Scoring system: 0: no staining ; 1+: staining of less than 25% of tumor cells; 2+: staining of 26% to 50% of tumor cells; 3+: staining of 51% to 75% of tumor cells; 4+: staining of more than 75%

of tumor cells.
